# Religious coping in the time of COVID-19 Pandemic in India and Nigeria: Finding of a cross-national community survey

**DOI:** 10.1177/0020764020984511

**Published:** 2020-12-27

**Authors:** Huma Fatima, Tosin Philip Oyetunji, Sudha Mishra, Krittika Sinha, Olorunyomi Felix Olorunsogbon, Oluwayemi Samson Akande, Srinivasan, Sujita Kumar Kar

**Affiliations:** 1 Department of Psychiatry, King George’s Medical University, Lucknow, Uttar Pradesh, India; 2 Faculty of Public Health, College of Medicine, University of Ibadan, Ibadan, Nigeria; 3 KGMU College of Nursing, King George’s Medical University, Lucknow, Uttar Pradesh, India; 4 Department of Health Promotion and Education, Faculty of Public Health, College of Medicine, University of Ibadan, Ibadan, Nigeria; 5 Department of Health Promotion and Education, College of Medicine, University of Ibadan, Ibadan, Nigeria

**Keywords:** Religion, religious coping, COVID-19, India, Nigeria

## Abstract

**Background:**

Religious and spiritual coping strategies is one of the possible tools that can be used to deal with stress and the negative consequences of life problems and illnesses. The study aims to assess religious coping in the time of the COVID-19 pandemic.

**Methodology:**

It was an online survey. The sample was collected using a snowball sampling technique as the data were collected through Google forms. The survey started on 22 April 2020 and was closed on 28 May 2020. The participants were from two countries, India and Nigeria. The inclusion criteria were age between 18 and 60 years, having completed at least 10 years of formal education, and have internet access. For data collection, Semi-structured proforma (demographic and personal characteristics) and Brief RCOPE was used to see the extent to which individuals engage in positive and negative forms of religious coping.

**Results:**

A total of 647 individuals (360 from Nigeria and 287 from India) participated in the survey. A total of 188 (65.5%) participants in India reported no change in their religious activities since they heard about COVID-19, while, 160 (44.4%) in Nigeria reported a decrease in religious activities. Positive religious coping in the Nigerian population was significantly higher than the Indian population. Similarly, negative religious coping was significantly higher (for most of the items in the brief RCOPE) in the Indian population than the Nigerian population.

**Conclusion:**

Significant percentages of people after the COVID-19 pandemic took religious coping steps to overcome their problems. During this pandemic, positive religious coping among the Indian and Nigerian communities is more prevalent than negative religious coping. There is a substantial cross-national difference between Indians and Nigerians in the religious coping modes.

## Introduction

Religion is an integral part of human civilization. It has a substantial impact on society and human behavior. Across the globe, people follow various religious beliefs. Religion acts as a double-edged sword, as it unites people as well as divides people. Religion influences coping positively and negatively (Pargament & Brant, 1998; Pargament & Park, 1997). The coping theories explain religion-coping connections. Religion also acts as a stress buffer to cope with the stress associated with illness (Siegel et al., 2001). Religion gives an explanatory model to combat stress-related to health issues. It, too, facilitates society's support system and strengthens the coping resources (Siegel et al., 2001). A study was done in patients with cystic fibrosis, and it was found that positive spiritual coping is associated with better health outcomes (Reynolds et al., 2014). An Indian study in remitted schizophrenia patients revealed that individuals using religious and spiritual coping adapt better to their life stress and illness-related stress (Das et al., 2018). Religious beliefs mitigate depression and suicidal behavior (Mosqueiro et al., 2015; Teismann et al., 2017). Positive religious beliefs help in strengthening trust, which helps in the development of resistance (Teismann et al., 2017). An Indian study compared religious coping of adolescents who attempted self-harm with healthy controls and found that positive religious coping was less frequently used by individuals with self-harm behavior (Grover et al., 2016). The same study also revealed that negative religious coping positively correlated with impulsivity and hopelessness (Grover et al., 2016). A study conducted in Nigeria estimated religious coping among people with depression and diabetes. It was found that older people with depression often have positive religious coping. Similarly, individuals from low socioeconomic status with diabetes have higher positive religious coping compared to individuals from higher socio-economic status (Amadi et al., 2016). These findings indicate that religious coping is primarily influenced by several socio-demographic characteristics.

Over the past several months, the COVID-19 pandemic has adversely affected the lives of people globally. During this pandemic, people experience anxiety, depression, and panic related to COVID-19, and there is a higher perceived mental healthcare need (Roy et al., 2020). During this pandemic, people adopt various measures to cope with their mental health challenges (Fullana et al., 2020). Various groups of the population possess varying degrees of vulnerability to acquire COVID-19. People differ in their belief systems and coping measures. During this COVID-19 pandemic, people commonly cope with stress by involving themselves in creative activities, involvement in recreational activities, exercise, online earning/learning activities, Yoga & meditation, and prayer. Failure of coping measures results in mental health issues (Kar et al., 2020). A recent study evaluated religious coping among the American orthodox Jewish population during this COVID-19 pandemic. In this study, it was seen that positive religious coping is associated with less stress, and negative religious coping is associated with more stress (Pirutinsky et al., 2020).

India is a country where people of different religious believes reside. Following religious rituals, cultural traditions, festivals, and religious ceremonies give a unique identity. Similarly, Nigeria is the most populated African country with cultural diversity. Both India and Nigeria struggle with the population explosion and scarcity of resources. These two countries fall in lower-middle-income countries as per the world bank (World Bank, 2020).

However, religion is often overlooked in researches involving cross-cultural psychology (Tarakeshwar et al., 2003). Many people have strong religious beliefs, and during challenging situations like the COVID-19 pandemic, religion may help people cope. Unfortunately, a paucity of research evaluated religious coping in the community population during this COVID-19 pandemic. Our study attempted to evaluate the religious beliefs and religious coping in the general population during the COVID-19 pandemic.

## Materials and methods

It was an online survey. The sample was collected using a snowball sampling technique as the data were collected through *Google forms*, in which people receiving the message were requested to complete the survey and then forward the link to their close contacts in various WhatsApp groups, Facebook, Email, and Twitter platforms. The survey started on 22 April 2020 and was closed on 28 May 2020. The participants were from two countries; India and Nigeria. The inclusion criteria were age between 18 and 60 years, having completed at least 10 years of formal education, and have internet access. The participation was completely voluntary. The participation was confirmed once the participants gave their consent for the same.

## Measures

### Semi-structured proforma (demographic and personal characteristics)

It included the name (voluntary), country, age, gender, education, occupation, marital status, religion, domicile, family type, either living alone or with family, and state/province of the participants’ current residence.

The investigators developed a questionnaire about one’s belief in the religion (believer or non-believer), about their performance of religious rituals (frequency), charity habits, and any change in these habits during the COVID-19 pandemic.

### The Brief RCOPE

It is a 14-item scale to see the extent to which individuals engage in positive and negative forms of religious coping. The Positive Religious Coping subscale assesses efforts to maintain a positive connection with God, collaborate with God, find positive meaning in the stressor, and let go of negative emotions. The Negative Religious Coping subscale assesses perceptions of a disrupted or conflictual relationship with God and one’s faith community, as well as a loss of faith in God’s power and belief that the devil caused the stressor (Pargament & Park, 1997). The respondents were asked to give a yes or no, as a response. It has good validity and reliability (Pargament et al., 2011).

The study proposal was reviewed and approved by the Institutional Ethics Committee of the study institute of India and Nigeria (India: IIIrd ECM COVID-19 IB/P3; Nigeria: AD 13/479/2000_B_).

Descriptive statistics were applied, and statistical analysis was done using the Graph Pad software online version. The mean values were compared by unpaired *t*-test, and the chi-square test compared the categorical variables. For statistical significance, the *p*-value was considered <.05.

## Results

During the survey, 670 responses were received, out of which 646 (96.06%) were consented and complete in all respects. The respondents’ age ranges from 18 to 60 years, whereas the Indian participants tend to be a little older with a higher mean age than Nigeria (32.30 vs. 28.30). Most of the participants, 399 (61.7%) from both countries, were male, which was higher among the Nigerian participants 225 (62.5%) compared to 174 (60.6%) in India (Table 1).

**Table 1.** Comparison between Indian and Nigerian participants on socio-demographic characteristics and religious coping behavior during COVID-19.

Variables	Total (*N* = 647)	India (*n* = 287)	Nigeria (*n* = 360)	*p*-value
Age (years) Mean ± *SD*	30 ± 7.58	32.30 ± 8.59	28.30 ± 6.21	*t* = 6.87; *p* < .001
Gender				
Male	399 (61.7%)	174 (60.6%)	225 (62.5%)	Chi-square: 0.237 *p* = .626
Female	248 (38.3%)	113 (39.4%)	135 (37.5%)	
Occupation				
Employed	366 (56.6%)	183 (63.8%)	183 (50.8%)	Chi-square: 39.38 *p* < .001
Unemployed	94 (14.5%)	19 (6.6%)	75 (20.8%)	
Students	166 (25.6%)	68 (23.7%)	98 (27.2%)	
House-wife	18 (2.8%)	15 (5.2%)	3 (0.83%)	
Others	3 (0.47%)	2 (0.7%)	1 (0.27%)	
Religion				
Hindu	218 (33.7%)	217 (75.6%)	1 (0.3%)	Chi-square: 492.04 *p* < .001
Muslim	112 (17.3%)	53 (18.6%)	59 (16.4%)	
Christian	306 (47.3%)	8 (2.8%)	298 (82.8%)	
Other	8 (1.2%)	7 (2.8%)	1 (0.3%)	
None	3 (0.47%)	2 (0.7%)	1 (0.3%)	
Domicile type				
Rural	96 (14.8%)	47 (16.4%)	49 (13.6%)	Chi-square: 9.975 *p* = .007
Semi-Urban	123 (19.0%)	39 (13.6%)	84 (23.3%)	
Urban	428 (66.2%)	201 (70.0%)	227 (63.1%)	
Type of family				
Joint	146 (22.6%)	103 (35.9%)	43 (11.9%)	Chi-square: 52.395 *p* < .001
Nuclear	501 (77.4%)	184 (64.1%)	317 (88.1%)	
Current living status				
Alone	190 (29.4%)	68 (23.7%)	122 (33.9%)	Chi-square: 8.017 *p* = .018
With family	449 (69.4%)	215 (74.9%)	234 (65%)	
Other	8 (1.2%)	4 (1.4%)	4 (1.1%)	
Do you believe in religion				
Yes	598 (92.4%)	251 (87.5%)	347 (96.4%)	Chi-square: 18.20 *p* < .001
No	49 (7.6%)	36 (12.5%)	13 (3.6%)	
Do you perform/offer your religious rituals				
Yes	464 (71.7%)	213 (74.2%)	251 (69.7%)	Chi-square: 1.745*p* = .418
No	112 (17.3%)	44 (15.3%)	68 (18.9%)	
Cannot say	71 (11.0%)	30 (10.5%)	41 (11.4%)	
How often do you perform your religion duties?				
Always	292 (45.1%)	96 (33.4%)	196 (54.4%)	Chi-square: 50.165 *p* < .001
Often	163 (25.2%)	76 (23.3%)	96 (26.7%)	
Sometimes	124 (19.2%)	81 (28.2%)	43 (11.9%)	
Rarely	42 (6.5%)	30 (10.5%)	12 (3.3%)	
Never	26 (4.0%)	13 (4.5%)	13 (3.6%)	
Do you observe any change in your religious activities since your heard about COVID-19? if yes, then in what way				
Increased	148 (22.9%)	61 (21.3%)	87 (24.2%)	Chi-square: 108.415 *p* < .001
Decreased	191 (29.5%)	31 (10.8%)	160 (44.4%)	
No change	293 (45.3%)	188 (65.5%)	105 (29.2%)	
Other	15 (2.3%)	7 (2.4%)	8 (2.2%)	
Do you offer charity?				
Yes	526 (81.3%)	222 (77.4%)	304 (84.4%)	Chi-square: 5.283 *p* = .021
No	121 (18.7%)	65 (22.6%)	56 (15.6%)	
Do you observe an change in charity pattern since you heard about the outbreak of COVID-19?, If yes then in what way				
Decreased	131 (20.2%)	14 (4.9%)	117 (32.5%)	Chi-square: 76.146 *p* < .001
Increased	232 (23.2%)	119 (41.5%)	112 (31.4%)	
No change	284 (43.9%)	154 (53.6%)	130 (36.1%)	
Responses to the items of brief rcope				
1. Looked for a stronger connection with God				
No	105 (16.2%)	76 (26.5%)	29 (8.1%)	Chi-square: 39.877 *p* < .001
Yes	542 (83.8%)	211 (73.5%)	331 (91.9%)	
2. Sought God’s love and care				
No	71 (11.0%)	58 (20.2%)	13 (3.6%)	Chi-square: 45.031 *p* < .001
Yes	576 (89.0%)	229 (79.8%)	347 (96.4%)	
3. Sought help from God in letting go of my anger				
No	166 (25.7%)	97 (33.8%)	69 (19.2%)	Chi-square: 17.922 *p* < .001
Yes	481 (74.3%)	190 (66.2%)	291 (80.8%)	
4. Tried to put my plans into action together with God				
No	94 (14.5%)	73 (25.4%)	21 (5.8%)	Chi-square: 49.41 *p* < .001
Yes	553 (85.5%)	214 (74.6%)	339 (94.2%)	
5. Tried to see how God might be trying to strengthen me in this situation				
No	99 (15.3%)	73 (25.4%)	26 (7.2%)	Chi-square: 40.874 *p* < .001
Yes	548 (84.7%)	214 (74.6%)	334 (92.8%)	
6. Asked for forgiveness of Sin				
No	100 (15.5%)	80 (27.9%)	20 (5.6%)	Chi-square: 60.877 *p* < .001
Yes	547 (84.5%)	207 (72.1%)	340 (94.4%)	
7. Focused on religion to stop worrying about my problems				
No	240 (37.1%)	152 (52.96%)	88 (24.44%)	Chi-square: 55.654 *p* < .001
Yes	407 (62.9%)	135 (47.04%)	272 (75.56%)	
8. Wonder whether God had abandoned me				
No	498 (77.0%)	206 (71.8%)	292 (81.1%)	Chi-square: 7.849 *p* = .005
Yes	149 (23.0%)	81 (28.2%)	68 (18.9%)	
9. Felt punished by God for my lack of devotion				
No	481 (74.3%)	210 (73.2%)	271 (75.3%)	Chi-square: 0.372 *p* = .542
Yes	166 (25.7%)	77 (26.8%)	89 (24.7%)	
10. Wondered what I did for God to punish me				
No	525 (82.2%)	219 (76.3%)	313 (86.9%)	Chi-square: 12.365 *p* < .001
Yes	115 (17.8%)	68 (23.7%)	47 (13.1%)	
11. Questioned God’s love for me				
No	525 (81.1%)	210 (73.2%)	315 (87.5%)	Chi-square: 21.43 *p* < .001
Yes	122 (18.9%)	77 (26.8%)	45 (12.5%)	
12. Wondered whether my church/temple/mosque had abandoned me.				
No	498 (77.0%)	206 (71.8%)	292 (81.1%)	Chi-square: 7.849 *p* = .005
Yes	149 (23.0%)	81 (28.2%)	68 (18.9%)	
13. Decided the devil made this happen				
No	499 (77.1%)	237 (82.6%)	262 (72.8%)	Chi-square: 8.694 *p* = .003
Yes	148 (22.9%)	50 (17.4%)	98 (27.2%)	
14. Questioned the power of God				
No	566 (87.5%)	223 (77.7%)	343 (95.3%)	Chi-square: 45.05 *p* < .001
Yes	81 (12.5%)	64 (22.3%)	17 (4.7%)	

Although employability significantly varies slightly between the Indian 183 (63.8%) and Nigerian 183 (50.8%) sample, most of the participants were employed.

Religious varies between both countries; the Hindu religion 218 (33.7%) was the most commonly reported practiced religion among Indian participants, whereas Christianity 298 (82.8%) was most frequent among the Nigerian study population. Both countries share a similar proportion in the practice of Islam religion.

More than half of the participants, 428 (66.2%), domiciled in the urban area and had a nuclear family system 501 (77.4%). Nigeria sample 317 (88.1%) exhibited a slightly higher proportion of nuclear family system than 64.1% reported in 184 India, whereas the percentage of urban dwellers was higher among Indian participants than Nigeria (70.0% vs. 63.1%). Most of the respondents, 598 (92.4%), believed in religion. However, there was a significant difference in religious belief between the two countries – Indian-251 (87.5%) versus Nigeria-347 (96.4%). On current living status, 449 (69.4%) of the participants lived with their families, with similar proportions observed in both countries.

Assessing the performance of religious rites, 213 (74.2%) in India and 251 (69.7%) in Nigeria offer religious rituals; of these, 96 (33.4%) and 196 (54.4%) do it always, respectively. Similarly, 188 (65.5%) participants in India reported no change in their religious activities since they heard about COVID-19, while, 160 (44.4) in Nigeria reported decreased.

Significantly, participants from both countries offer charity, 222 (77.4%) in India and 304 (84.4%) in Nigeria, whereas two-fifth (*n* = 119, 41.5%) in India reported increased charity pattern since the heard of COVID-19 outbreak compared with 112 (31.4%) observed among Nigeria participants. Conversely, a notably decreased charity pattern during the outbreak was higher among participants in Nigeria (*n* = 117, 32.5%) than India (*n* = 14, 4.9%). In looking for a more robust connection with God, 211 (73.5%) participants in India and 331 (91.1%) in Nigeria sought a stronger with God while a similar proportion of these participants from each country 229 (79.8%) and 347 (96.4%) sought God’s love and care, respectively. There was a notable difference between both countries’ responses when asked if help was sought from God for anger management. In India, 190 (66.2%) did seek God to let go of anger compared to 291 (80.8%) among participants in Nigeria.

The majority of respondents, (*n* = 339, 94.2%), (*n* = 334, 92.8%), and (*n* = 340, 94.4%) in Nigeria, tried to put plans into action together with God, tried to see how God might strengthen them and asked for forgiveness during the outbreak respectively. However, these proportions were lower (*n* = 214, 74.5%), (*n* = 214, 74.6%), and (*n* = 207, 72.1%) among Indian participants. In both countries’ response to focusing on religion to stop worrying about problems, 272 (75.56%) in Nigeria focused on religion to stop worrying about a problem; In contrast, the proportion was lower among India sample 135 (47.04%).

Further, a higher proportion in India participants – 81 (28.2%) and 77 (26.8%) wondered whether God had abandoned them and felt punished by God for lack of devotion compared to 68 (18.9%) and 89 (24.7%) in Nigeria respectively. Besides, 77 (23.7%) participants in India and 47 (13.1%) in Nigeria wondered what they had done to be punished by God. In questioning God’s Love and power, a higher proportion was significantly observed in India; 77 (26.8%) and 64 (22.3%) reported to have questioned God’s love and power compared to 45 (12.5%) and 17 (4.7%) in Nigeria, respectively. However, a higher proportion of the participants in Nigeria, 98 (27.2%) believed the devil made the outbreak happen while 50 (17.4%) in India (Table 1).

## Discussion

The impact of COVID-19 on society is enormous. COVID-19 pandemic resulted in significant mortality and morbidity worldwide. The devastating effect of the COVID-19 pandemic affected the general well-being, including mental health. During this challenging situation, people have difficulty coping with anxiety, depression, fear, stress, and losses (Kar et al., 2020). Confronted with ambiguity and adversity, humans prefer to use religion to offer comfort and explanation. People practice religion to deal with stressful and distressing incidents in life for which they are not prepared enough (Sinding Bentzen, 2019).

Previous studies showed that natural disasters have a long-lasting effect on religiosity spread across generations (Sinding Bentzen, 2019). Whether the COVID-19 pandemic will have similar long-term effects is yet to be seen. Our study had two catchment zones (India and Nigeria), where religious beliefs differ. The population characteristics of the two zones also differ, which may influence the religious coping. Our study population is significantly different in India and Nigeria in several socio-demographic characteristics (e.g. more unemployed in Nigeria, Hindu prominence in India and Christian prominence in Nigeria, more joint family system in India, living alone more in Nigeria). There is a significant difference in religious belief in the Nigerian population than India (Nigeria 96.4% vs. India 87.5%). The belief in religion may influence religious coping behavior. Individuals with more belief in religion may adopt religious coping to combat their stress than those who had less belief in religion. It may be supported by the hypothesis, accessibility to coping resources determine the selection of coping strategy (Pargament & Park, 1997; Siegel et al., 2001). In India and Nigeria, approximately 70% of individuals do religious rituals, and more than half do religious duties more often. In Nigeria, there is a decline in religious activities in >44% of participants during the COVID-19 pandemic (vs. India 10.8%). It might be due to differences in preference to offer prayer at religious places. The majority of Indians offer prayer in their home settings, which might be why there is little decline in religious activities. During the COVID-19, pandemic charity behavior in the Indian population increased significantly than the Nigerian population. These are the religious ways to get satisfaction and help in releasing guilt and shame. It has been seen that during crisis situations, people often indulge in religious activities, which was also reported during this COVID-19 pandemic (Bentzen, 2020). During this COVID-19 pandemic, worldwide people pray to end the crisis. It has been reported in people of all socio-economic strata (Bentzen, 2020). In a community survey during the COVID-19 pandemic in India, more than half of the participants agreed that there is an increase in spirituality during the ongoing pandemic among the general population (Tripathy et al., 2020). Our study found that positive religious coping in the Nigerian population was significantly higher than the Indian population. Similarly, negative religious coping was significantly higher (for most of the items in the brief RCOPE) in the Indian population than the Nigerian population. Evidence supports that positive religious coping helps combat stress, whereas negative religious coping may worsen stress and guilt (Pirutinsky et al., 2020). During the study period, the number of COVID-19 cases and mortality was higher in India than in Nigeria. As per the WHO situation report-99, on 28 April 2020, the total number of COVID-19 cases in India and Nigeria was 29, 435, and 1337, respectively. At the same time, the number of deaths in India and Nigeria was 934 and 40, respectively (World Health Organization [WHO], 2020). As the number of cases and mortality was higher in India, it might be possible that people may change from positive religious coping to negative religious coping when the situation is getting worse. Approximately 1/4th to 1/3rd of the general population during this COVID-19 pandemic experience stress, anxiety, or depression (Krishnamoorthy et al., 2020; Salari et al., 2020). However, a paucity of research estimated anxiety, depression, and stress due to COVID-19 in Nigeria. It is worth studying religious coping concerning mental health outcomes like anxiety, depression, and stress. It may give insight into the role of religious coping in preventing adverse mental health outcomes. Earlier evidence suggests that positive religious coping is associated with a better overall quality of life in cancer patients (Tarakeshwar et al., 2006). It can be hypothesized that positive religious coping may also improve individuals’ quality of life experiencing psychological distress. There is a need for religious and spiritual optimism to counter the ongoing distress due to the COVID-19 pandemic.

## Limitations

Small sample size and cross-sectional study designs are significant limitations of the study, limiting the generalizability. As snow-ball sampling was done, there is a possibility of referral bias. Another limitation being, use of questionnaires in the English language in an online platform. A face-to-face interview technique using a regional language questionnaire may overcome the limitations by addressing diverse groups of people.

## Conclusion

During the COVID-19 pandemic, a significant number of people adopt religious coping measures to combat their difficulties. Positive religious coping is more common than negative religious coping among the Indian and Nigerian populations during this pandemic. There is significant cross-national variation in the religious coping styles between Indians and Nigerians.
